# Plague Vaccine Development: Current Research and Future Trends

**DOI:** 10.3389/fimmu.2016.00602

**Published:** 2016-12-14

**Authors:** Shailendra Kumar Verma, Urmil Tuteja

**Affiliations:** ^1^Microbiology Division, Defence Research & Development Establishment, Gwalior, India

**Keywords:** plague, *Yersinia pestis*, molecular adjuvants, subunit vaccines, lethal disease, cellular immunity, DNA vaccines, live delivery

## Abstract

Plague is one of the world’s most lethal human diseases caused by *Yersinia pestis*, a Gram-negative bacterium. Despite overwhelming studies for many years worldwide, there is no safe and effective vaccine against this fatal disease. Inhalation of *Y. pestis* bacilli causes pneumonic plague, a fast growing and deadly dangerous disease. F1/LcrV-based vaccines failed to provide adequate protection in African green monkey model in spite of providing protection in mice and cynomolgus macaques. There is still no explanation for this inconsistent efficacy, and scientists leg behind to search reliable correlate assays for immune protection. These paucities are the main barriers to improve the effectiveness of plague vaccine. In the present scenario, one has to pay special attention to elicit strong cellular immune response in developing a next-generation vaccine against plague. Here, we review the scientific contributions and existing progress in developing subunit vaccines, the role of molecular adjuvants; DNA vaccines; live delivery platforms; and attenuated vaccines developed to counteract virulent strains of *Y. pestis*.

## Introduction

Plague is a historic disaster which has caused ~200 millions of deaths during the pandemics (1). It is an enzootic disease and prevalent in many parts of the world, with the organism being transmitted through infected fleas from rodent reservoirs to humans (2). Human is an accidental host and can carry bubonic plague if contacted by a flea containing plague bacilli. Bubonic plague can develop into septicemic plague or a secondary pneumonic plague if not treated in time. Besides, plague can also be contracted by direct transmission through aerosols to cause an extremely lethal form of primary pneumonic plague. *Yersinia pestis* exhibits intrinsic genetic plasticity (3, 4), can attain antibiotic resistance (5–8), and has been used as a biowarfare agent (9–11). Therefore, Centers for Disease Control has listed *Y. pestis* under the category A select agent.

To date, there is no approved vaccine against plague in the developed world, a live vaccine made in 1920s, has been used by many countries for immunization (12). Early diagnosis can help in the treatment of plague patients with antibiotics; however, there are few reports which confirm the existence of antibiotic resistance strains of *Y. pestis* (5, 6). Recently, naturally harboring multidrug resistance variants of *Y. pestis* have been isolated in Mongolia (13). The whole genome sequencing studies showed very less difference between the current circulating strain of *Y. pestis* and the strain responsible for fourteenth century pandemic (14). Moreover, it is quite evident that *Y. pestis* can be converted into a multidrug-resistant strain by genetic manipulations in the laboratory (6, 15). Taking into account of these factors, i.e., rapid progression of the disease and 100% mortality rate of pneumonic plague, a potential biowarfare agent and the emergence of multidrug resistant variants of plague microbe make imperative to develop an ideal and effective vaccine against this highly fatal disease.

## Concept to Develop Plague Vaccines

*Yersinia pestis* suppresses the immunity and survives in susceptible hosts, but this capability of the pathogen cannot be applied on infection-survived animals because their immune system resists the re-infection (16). This specific skill of the host to defend against re-infection opened up the opportunities and new avenues to develop vaccine/s to confer protection against this lethal disease.

## Whole-Cell-Based Vaccines Against Plague

The idea to develop vaccine against plague started by Alexandre Yersin in 1895 who investigated immunity against *Y. pestis* in small animal models in his laboratory. He evaluated heat-killed whole-cell vaccine, attenuated live strains of *Y. pestis*, by immunization in animals with repeated boosters (17). These findings encouraged researchers to develop two types of vaccines, i.e., killed whole cell (KWC)- or live whole cell (LWC)-based vaccines modified from virulent strains of *Y. pestis*. To prepare the KWC vaccine, *Y. pestis* bacilli were inactivated either by heating or using chemicals. These vaccines were found safe and evoked immunity against bubonic plague but found inefficient against pneumonic plague in primed animal models (18). Later, Meyer and colleagues developed a more advanced formalin-killed whole-cell vaccines (19, 20). A vaccine (USP) developed by this method was approved in USA. Human immunization with formalin-killed, whole-cell vaccine during the Vietnam War indirectly proved that this vaccine protects against bubonic plague (19, 21). On the other hand, this vaccine was not only highly reactogenic and inefficient to provide long-term protection but also fail to protect against pneumonic plague (19, 20, 22, 23). Therefore, these killed whole-cell-based vaccines are not appreciable for use against biothreat scenario.

Live whole cell-based vaccines were prepared from fully virulent strains of *Y. pestis* after multiple passages. These types of vaccines were able to induce strong immune response against both types of plague: bubonic and pneumonic. But there is always a risk associated with these vaccines regarding the ability of live bacilli to colonize and temporarily replicate in host. Many fatal cases were seen in laboratory animal models and in non-human primates (NHPs), after vaccination with live vaccines (19, 20). However, there was no fatal human case reported after administration of LWC plague vaccine for many years. Even though, millions of people were vaccinated with the LWC in the middle of twentieth century (24), the countries of the former Soviet Union and China are still using LWC-based vaccine against plague for human vaccination. The potential of LWC-based vaccines have been confirmed in humans for many years; however, these vaccines are associated with several adverse effects, and leg behind to provide long-term immunity (12, 25).

## Strategies for the Development of Subunit Plague Vaccine

With the advent of recombinant DNA technology, immunodominant and protective antigens can be easily identified and prepared in purified form for the development of subunit vaccines. Most importantly, these subunit vaccines reduced the risk factors and adverse effects associated with live and KWC vaccines. However, thorough clinical trials are compulsory to confirm that these vaccines are superior and safe in comparison to whole-cell-based vaccines.

Mainly two virulent factors, capsular F1 and the low calcium response LcrV antigens of *Y. pestis*, have been demonstrated by various researchers throughout the world and proven to be the best to provide protection in various animal models. Immunization with recombinant F1 imparts same degree of protection in mice against subcutaneous or pneumonic plague as does native F1, extracted from *Y. pestis* (26). In our studies (27), vaccination with recombinant F1 failed to protect mice against bubonic plague. Nevertheless, there exist some virulent strains of F1-negative *Y. pestis*; hence, vaccines based exclusively on F1 are not worthwhile against any type of plague (28). In 2011, Chopra’s group generated Δcaf mutant *Y. pestis* in the laboratory by homologous recombination and proven the virulence in a mouse model. The Δcaf mutant was observed as virulent as WT CO92 in the pneumonic plague (29). In case of LcrV, immunization with both native purified and recombinant LcrV provides protection in mice against bubonic and pneumonic plague (30, 31). Rabbit polyclonal IgG against an engineered fusion peptide, PAV, provided excellent passive immunity (100% protection) against intravenous (i.v.) challenge of *Y. pestis* and *Yersinia pseudotuberculosis* in Swiss Webster mice (32, 33). We also reported that recombinant LcrV alone provided only 75% protection in mice against bubonic plague (27). The combination of recombinant F1 and LcrV antigens elicited greater protection in comparison to either F1 alone or LcrV alone (34, 35). Vaccination with F1 and LcrV antigens adjuvanted with alum protects mice against pneumonic plague, proven by The United Kingdom’s defense department (36, 37). The United States Army Medical Research Institute of Infectious Diseases demonstrated that a recombinant bivalent F1–LcrV fusion protein provides protection in mice challenged *via* aerosolized route against virulent strains of *Y. pestis* (26, 38). In a recent study, F1mut-V in formulation with alhydrogel and T4-decorated F1mut-V without any adjuvant imparted 100% protection in mice and rats against pneumonic plague (39). In our recent studies, we also demonstrated that a mixture of recombinant F1 + LcrV antigens in formulation with alum imparts full protection in mice against bubonic plague (27). In conclusion, F1/LcrV vaccine provides strong protective immunity in mice, rats, and rabbits against subcutaneous and pneumonic plague. The vaccine mainly induced humoral immune response as a high titer of anti-LcrV antibodies is very crucial. The protection is stimulated by anti-LcrV antibodies, which help by blocking the type 3 secretion system (40, 41). Inclusion of F1 with LcrV enhanced the protection as LcrV does not always provide 100% protection (27).

Due to the ethical limitations, it is not possible to challenge human with *Y. pestis*; hence, the NHPs or monkeys are considered as standard model for trials of plague vaccines. The intramuscular vaccination of F1/V vaccines in formulation with alhydrogel protects cynomolgus macaques against pneumonic plague (42, 43); however, this F1–LcrV vaccine provided poor and inconsistent (0–75%) protection in African green monkeys (44–46). The F1/LcrV vaccine also provided protection against subcutaneous challenge of virulent *Y. pestis* in rhesus macaques (47) and baboons (48). The existence of F1-negeative virulent strains of plague *bacilli* (12, 49) and LcrV variants of *Y. pestis* (50, 51) may not be ignored to not to confer the cross protection. The subunit vaccine exclusively based on F1/LcrV may not be worthwhile for a biothreat scenario, hence, the inclusion of additional subunits to the F1/LcrV vaccine is utmost needed. Vaccination with recombinant YscF in formulation with Freund’s adjuvant protected mice from an i.v. challenge with *Y. pestis* (52). In another study, immunization with recombinant YscF in formulation with Ribi adjuvant system R-730 monophosphoryl lipid A provided significant protection in mice against subcutaneous challenge with *Y. pestis* (53). In a recent study, anti-Ail/OmpX and anti-OmpA antibodies protected mice against bubonic plague, and anti-Pla antibodies were protective against pneumonic when challenged with the F1-negative CO92 strain (54). In the past, a number of antigens have been evaluated for protection with very little success (45).

## Role of Molecular Adjuvants

The ultimate goal of vaccination is to stimulate a strong and long-lasting immunity to the administered subunit candidate against infection. Generally, the problem associated with purified recombinant subunit vaccine candidates is the poor/less-induced immunogenicity in comparison to LWC- or KWC-based vaccines. To deal with this problem, alum is the one and only approved human compatible adjuvant and has been used for human vaccination widely, but alum mainly stimulates the Th2 response. The important facts to use adjuvants are (1) to augment the immune response of purified recombinant subunits, (2) to improve the protective potential, (3) to decrease the amount of dose and number of vaccination, and (4) to help in delivery of an antigen to the target cells (55–57).

The immunization of mice with F1 and LcrV antigens in formulation with molecular adjuvants, i.e., cholera toxin and heat-labile enterotoxin (LT), both formulations provided partial protection against 100 MLD against a virulent strain of *Y. pestis* (58). Flagellin, agonist of toll-like receptor-5, is mostly used molecular adjuvant with F1–LcrV to augment the Th1 type of immune response and provided 100% protection in mice against a respiratory challenge with *Y. pestis* (46, 59) and showed variable protective efficacy in NHPs against pneumonic challenge (46). CpG Oligodeoxynucleotides, agonist of TLR-9, elicit a balance Th1/Th2 response in formulation with F1–LcrV fusion protein and protected mice against bubonic and pneumonic plague (60, 61). Microencapsulation (62) and lipid A mimetics (63) of recombinant LcrV–F1 fusion protein induced a mixed Th1/Th2 cell-mediated immune response and provided protection against pneumonic plague in mice and rats. Liposome, such as cationic liposome nucleic acid complexes (64), and proteosomes, such as protollin (65), have been evaluated with F1–LcrV and have been shown to protect against pneumonic plague.

In 2013, Tao et al. addressed a series of concerns and generated mutants of F1 and V, which are completely soluble and produced in high yields. The authors engineered the vaccine into a novel delivery platform using the bacteriophage T4 nanoparticle. The nanoparticle vaccines induced strong immune response and conferred 100% protection against pneumonic plague in mice and rats (39). Some novel agonist of the costimulatory molecules of tumor necrosis factor receptor super family has been shown to stimulate T cell activation, expansion, and acquisition of effector function. One such molecule, SA-4-1BBL (recombinant agonist of 4-1-BB costimulatory molecule), had shown a better efficacy in generating CD4^+^ and CD8^+^ T cells producing TNF-α and IFN-γ with F1–LcrV fusion protein and provided 100% protection against bubonic model of plague in C57BL/6 mice (66). Addition of HSP70 (domain II) of *Mycobacterium tuberculosis* with F1/LcrV subunits of *Y. pestis* augments the cellular immune response. HSP70(II) significantly elevated the levels of IL-2, IFN-γ, TNF-α, and IFN-γ secreting CD4^+^/CD8^+^ T cells in F1 + LcrV + HSP70(II) vaccinated group in comparison to the F1 + LcrV group. F1 + LcrV + HSP70(II) combination provided full protection against virulent strain of *Y. pestis* (S1, Indian clinical isolate) in a mouse model (27). Later, we designed a recombinant trivalent fusion protein F1–LcrV–HSP70(II) and evaluated in a mouse model. This trivalent fusion protein provides improved cellular immune response and full protection against plague (67). Overall, the molecular adjuvants play the crucial role in vaccine development from antigen delivery to augmentation of the immune response in the host.

## DNA Vaccines Strategies

Attempts were made to develop recombinant DNA vaccines against plague. Initially, the success rate was low as generating weak humoral immune response to F1 and LcrV. The LcrV/F1-based DNA vaccines were developed as an alternative approach to protein-based vaccines that contain either full or part of the open reading frames encoding LcrV, F1, or both. A peptide (127-amino acid) vaccine of LcrV antigen elicited a strong humoral immune response and provided 60% protection against *Y. pestis* in mice (68). The addition of the constructs of molecular immunopotentiator IL-12 with F1 or LcrV has significantly enhanced the immune response and showed 80% protection from a subsequent inhalational challenge with *Y. pestis* (69). The DNA constructs were prepared to express the *Y. pestis* antigens with human tissue plaminogen activator (tPA) signal sequence to get the secretory proteins in absolutely soluble forms. DNA vaccination of LcrV with tPA elicited a significant humoral immune response and protected against pneumonic plague (70). In a recent study, DNA vaccine of LcrV elicited a robust CD8^+^ T cell immune response against specific epitopes (71). Some other vaccine candidates, i.e., YscF, Pla, YopB, YopD, and YpkA, were also evaluated in small animals and showed inadequate protection (72). In conclusion, the result of DNA vaccination was highly reliant upon the DNA vaccine construct, and this technology must be encouraged and optimized for future use of human vaccination against plague.

## Live Carrier Platforms for Vaccine Delivery

Apart from the testing of plague subunit vaccines, the expression of vaccine candidates from *Y. pestis* in live carriers also started. These types of systems have their own limitations, because the expertise of attenuation is needed when virulent genes are expressed as protective antigens. Attenuation is an important step because a huge risk is associated particularly for immunocompromised populations. It is very important to know that how to prepare and store the vaccine stockpile in order to make sure the viability of the vector for delivery. Nevertheless, the advantage of the live vaccines is the low cost, easy to scale-up the production, and the potential to induce strong cell-mediated and mucosal immune response.

## Live Viral-Based Delivery Platforms

Replication-deficient adenoviral vectors (Ad) are well-established delivery platforms as they transfer gene/s efficiently to the macrophages following their activation, and therefore evoking robust and fast humoral and cellular immunity. An adenovirus (Ad) gene-transfer vector encoding LcrV vaccine candidate was developed by Crystal’s group (73, 74). Single immunization with this recombinant virus was found efficient to induce strong humoral and cellular immunity and provided protection against pneumonic plague in mice (73, 74). Later, they linked either LcrV or F1 vaccine candidate to the capsid protein pIX of adenovirus and demonstrated that both constructs induced significantly robust IgG response in the sera of intramuscular (i.m.)-vaccinated mice in comparison to the vaccination with purified LcrV/F1 in formulation with adjuvant (73, 74). Recently, a replication-defective human type 5 adenovirus (Ad5) vector was used to construct the recombinant monovalent and trivalent vaccines (rAd5-LcrV and rAd5-YFV). The monovalent codon-optimized *lcrV* gene expressed LcrV and the trivalent fusion gene-designated *YFV* expressed a trivalent fusion protein YscF–F1–LcrV. Vaccination of mice with the trivalent rAd5-YFV construct provided superior protection in comparison to monovalent rAd5-LcrV construct against bubonic and pneumonic when challenged *via* either the i.m. or the intranasal (i.n.) route. Immunization of cynomolgus macaques with the trivalent rAd5-YFV provided 100% protection against pneumonic plague. This has first ever proved the efficacy of an adenovirus-vectored trivalent rAd5-YFV vaccine against pneumonic plague in mouse and NHP models (75).

One more vaccine based on recombinant vesicular stomatitis virus vectors harboring the gene encoding LcrV antigen was developed and evaluated in mice by Rose’s group (76, 77). The genes encoding LcrV and F1 were cloned in Vaccinia viral-based vectors and evaluated in Balb/C mice. The observed response was significantly immunogenic, and vaccine was found safe in immunocompromised SCID mice (78, 79). It was reported in a study that latent infection with either murine gammaherpesvirus 68 or murine cytomegalovirus in mice provides protection against *Listeria monocytogenes* or *Y. pestis* challenge *via* both routes either intranasal or subcutaneous. The mechanism of protection was the long-lasting expression of interferon-γ and activation of antigen-presenting cells, which evoked the innate immunity against challenge of *Y. pestis* (80). The scientists of National Wildlife Health Center, USA, evaluated Racoon poxvirus (RCN)-based two vaccine constructs, RCN-F1 and RCN-V307. The consumption of baits containing both vaccine constructs, i.e., RCN-F1 and RCN-V307, by prairie dogs (*Cynomys ludovicianus*) showed significant protection against plague challenge (81). Later, the same group evaluated a dual RCN-F1/V307 construct that expresses both F1 and V307 antigens. The RCN-F1/V307 vaccine imparted similar degree of protection against plague not only in mice but also in prairie dogs as compared to single antigen constructs. The RCN-F1/V307 vaccine also provided protection in mice against an F1-negative strain of *Y. pestis* (82, 83).

## Live Bacterially Based Delivery Platforms

An attenuated *Salmonella* was used first ever as a live delivery platform to express F1 and LcrV antigens. In orally vaccinated animals, these vaccines induced the expression of IgG2a subtypes and found inefficient to protect against plague. Immunization *via* nasal route and boosting with purified antigens *via* parenteral could be the better approach to augment the immunity. Still, the protection was significantly less in comparison to protection provided by immunization with recombinant antigens (49). Several studies have been performed by Titball’s group; they designed and prepared to express the bivalent F1–LcrV fusion protein (84), LcrV only (85), and F1 on cell surface (86). A *Salmonella* strain was constructed to express F1 as an extracellular capsule and soluble LcrV antigen (87). Largely, *S*. Typhimurium strains expressing F1/LcrV or truncated LcrV were performed to evoke the IgG- and cell-mediated immune response to the protein of interest. These vaccines provide partial protection against *Y. pestis* infection *via* nasal or subcutaneous route.

Morton et al. studied a *S*. Typhi strain expressing F1 provided protection in mice after intranasal vaccination against plague (88). One similar type of vaccine strain was also performed in 7-day-old mice after immunization *via* intranasal route (89). The vaccinated animals developed mucosal immune response, and IFN-γ-expressing cells and were primed with F1 in formulation with alum. This *Salmonella* vaccine offered strong priming in comparison to F1 with alum prime, proving the better efficiency of *Salmonella*-based vaccine for plague in a prime boost setting. Newly invented vectors derived from *Salmonella* with better immunogenicity, controlled mechanism of attenuation, and long-term stability in expression of antigens of interest has endorsed the improved protective potentials of vaccine mainly based on the tailored LcrV (90). The degree of immunity of this vaccine is not as good as induced by the purified F1 and LcrV vaccines. However, *Salmonella*-vectored system is quite appropriate to express the outer membrane proteins, i.e., PsaA, adhesin, and HmuR, which have been evaluated as vaccine candidates (91, 92). There is one more report where LcrV antigen from *Y. pseudotuberculosis* was expressed in a heterologous delivery system; the commensal, non-pathogenic species *Lactococcus lactis*. This *L. lactis* expressing LcrV did not protect against *Y. pestis*; however, it protected well against *Y. pseudotuberculosis* (93). In a recent study, intranasal immunization of mice with *L. lactis* secreting LcrV antigen from *Y*. pseudotuberculosis (Ll-LcrV) induced strong humoral and cellular immunity against *Y. pseudotuberculosis* infection in a mouse model and provided a significant long-lasting protection (94).

A remarkable progress has been made by Carniel’s group on developing plague vaccine using avirulent *Y. pseudotuberculosis*. Vaccination against bubonic plague can be obtained using attenuated *Y. pseudotuberculosis* (strain IP32680). One oral IP32680 inoculation provided 75% protection and two inoculations provided 88% protection against subcutaneous challenge with *Y. pestis* CO92 in a mouse model (95). The similar group generated an encapsulated *Y. pseudotuberculosis* by cloning the F1 encoding *caf* operon of *Y. pestis*. A single oral vaccination with the live attenuated *Y. pseudotuberculosis* V674pF1 imparted significant protection against pneumonic plague in mice (96). Later, the *caf* operon was manipulated into the chromosome of a genetically attenuated *Y. pseudotuberculosis*, yielding the VTnF1 strain. A single dose by oral administration of VTnF1 vaccine provided 100% protection against both bubonic and pneumonic plague in mice. The authors claimed that VTnF1 strain is easy-to-produce, genetically stable, and irreversibly attenuated and provides long-lasting immunity against both wild-type and F1-negative *Y. pestis* (97, 98).

## Attenuated Plague Vaccines

An attenuated vaccine is created by reducing the virulence of a pathogen, but still keeping it live. Attenuation takes an infectious agent and alters it so that it becomes avirulent. In 1936, a human live plague vaccine developed from an attenuated strain EV NIIEG of *Y. pestis* has been extensively used in Russia. This is a subculture of vaccine strain EV76, developed at the NIIEG (Scientific-Research Institute for Epidemiology and Hygiene, Kirov, Russian Federation) in the former USSR (17, 99). EV NIIEG is the only approved vaccine against plague for human use during plague outbreaks. The short-term immunity and the concern of safety are the limitations of this vaccine. Additionally, LPV provides poor protection to mice against non-encapsulated *Y. pestis* challenge (25). The vaccination showed several side effects due to its highly toxic nature. The vaccine EV NIIEG was modified by removing the *lpxM* gene for the late acyltransferase, resulting in formation of predominantly less-toxic penta-acylated lipid A. The modified mutant vaccine conferred better protection in mice and guinea pigs due to the optimal expression of protective antigens and its long-lasting existence in immunized animals (100, 101). Another approach used by *Y. pestis* expresses tetra-acylated lipid A that can not activate TLR-4 so that the pathogen can breach the host immune system. This is happened because both the *lpxP* and *lpxM* genes expresses hexa-acylated lipid A at 28°C. Both LpxP and LpxM are important for hexa-acylation. But at 37°C, in human host, *lpxP* is not stimulated to express, which results in tetra-acylated lipid A. TLR-4 does not recognize tetra-acylated lipid A (102). TLR-4 recognizes hexa-acylated lipid A (103–105). Researchers introduced the *lpxL* gene of *E. coli* expressing acyltransferase absent in *Y. pestis*. The engineered strain constitutively expressed hexa-acylated lipid A that evokes the innate immune system at the early hours of *Y. pestis* infection leading to its elimination (106).

Another successful strategy is to attenuate the wild-type strain of *Y. pestis* to develop the vaccine. Therefore, the attenuated strains carrying the mutations in the *lpp* and *nlpD* genes encoding outer membrane lipoproteins showed outstanding capability to induce protective immune response against *Y. pestis* (107, 108). A deletion in the global regulator gene *rovA* of *Y. pestis* was introduced and tested as a vaccine candidate (109). Likewise, a number of mutations in wild-type strain of *Y. pestis* were introduced to diminish the virulence. Many mutant strains were evaluated for their protective efficacy, which were having the mutations in the genes, i.e., *yopH* encoding effector protein of type 3 secretion system; *aroA* gene for aromatic-dependent; *guaB* gene encoding for guanine nucleotide bio-sysnthesis; *crp* gene encoding for cyclic adenosine monophosphate receptor protein; *relA* and *spoT* genes; *smpB-ssrA* genes encoding housekeeping functions for the translational machinery; and *dam* gene for DNA adenine methylase (92, 110).

## Future Perspective

The F1/LcrV-based subunit vaccine mainly induces a humoral immune response. While this vaccine has shown promising results in animal models, its protective potential in humans is yet to be assessed. In the near future, the F1/LcrV-based vaccine may be accessible to populations residing in plague-endemic areas. Since the protective effect of this vaccine is mainly dependent on humoral immunity, it may be essential to administer boosters from time to time. Next-generation plague vaccines should be designed to stimulate strong cell-mediated immunity as well. Both of the responses, humoral and cellular, effectively contribute to vaccine efficiency. The humoral immune response refers to the production of antibodies that neutralize extracellular microbes, while the cellular immune response relies on T cells which express cytokines and actively destroy intracellular microbes. The heat-shock protein 70 (domain II) of *M. tuberculosis* and SA-4-1BBL (recombinant agonist of 4-1-BB costimulatory molecule) modulate cellular immunity and have been used to enhance the protective potential of F1/LcrV antigens in mice; however these studies need further evaluation in NHPs (27, 66).

In USA, existing KWC or live attenuated vaccines against plague are presently not preferred due to ethical concerns. As a result, a prime boost strategy to improve the protective capacity of next-generation DNA or live carrier vaccines would be of immense interest (92, 110). Reasonably, live attenuated *Y. pestis* strains stimulated complete protection against plague in animal models. Current live *Y. pestis* vaccines must stimulate both humoral- and cell-mediated immunity to a range of important antigens, imparting robust protection, especially in comparison to the subunit vaccines. Thus, there is an urgent need to extend research for the development of new innovations and improved version of live attenuated vaccines.

## Author Contributions

SV collected the literature and prepared the manuscript, and UT reviewed and edited the manuscript.

## Conflict of Interest Statement

The authors declare that the research was conducted in the absence of any commercial or financial relationships that could be construed as a potential conflict of interest.
